# Vacancy notice for the post of Director of ECDC, Stockholm, published

**DOI:** 10.2807/1560-7917.ES.2016.21.41.30369

**Published:** 2016-10-13

**Authors:** Eurosurveillance editorial team

**Affiliations:** 1 European Centre for Disease Prevention and Control (ECDC), Stockholm, Sweden

**Keywords:** public health

The Director is the legal representative and public face of the European Centre for Disease Prevention and Control (ECDC) and is accountable to the ECDC Management Board. She/he will lead and manage ECDC and take overall responsibility for its operations, ensuring the achievement of ECDC’s objectives.

For detailed information on the job description, requirements and the application procedure, please visit the ECDC website at: http://ecdc.europa.eu/en/aboutus/jobs/Pages/JobOpportunities.aspx

The deadline for applications is 9 November 2016 at 12:00 noon Brussels time.

